# Special Issue: Juvenile Onset Huntington’s Disease

**DOI:** 10.3390/brainsci10090652

**Published:** 2020-09-20

**Authors:** Peg C. Nopoulos

**Affiliations:** 1 Department of Psychiatry, Carver College of Medicine at the University of Iowa, Iowa City, IA 52242 USA; Peggy-Nopoulos@uiowa.edu; Tel.: +1-319-356-1144; 2 Department of Neurology, Carver College of Medicine at the University of Iowa, Iowa City, IA 52242, USA; 3 Department of Pediatrics, Carver College of Medicine at the University of Iowa, Iowa City, IA 52242, USA

The Special Issue “Juvenile Onset Huntington’s Disease” highlights the growing interest in understanding the unique aspects of this ultra-rare disorder. Adult Onset Huntington’s Disease (AOHD) is a single gene disorder caused by a triplet repeat expansion in the Huntingtin gene. With decades of research to support the search for a cure, we are now in an exciting time of true progress in fighting AOHD with gene therapy trials underway. However, excluded from current studies are the subset of patients who, by virtue of very high CAG repeat expansion (typically over 60), have onset of disease early in life, defined by motor onset prior to age 21 and referred to as Juvenile Onset Huntington’s Disease (JOHD). This definition is somewhat arbitrary as the pathogenic mechanism is exactly the same—expanded CAG repeat in the Huntingtin gene. Nevertheless, due to its rarity, there is a relative dearth of studies on JOHD, leaving many questions regarding its phenomenology.

The current issue includes seven articles that span a variety of topics including the difficult emotional experience that parents endure in the context of their child becoming ill and diagnosed with JOHD [1]; a review of the clinical manifestations of JOHD [2]; and four articles from the only prospective study of JOHD evaluating behavior [3], the association of CAG repeat and motor onset [4], autonomic nervous system dysfunction [5], and abnormality in the unique MRI marker of T1rho in JOHD subjects [6]. Finally, this issue is rounded out by a review of the therapeutic advances for HD, highlighting the possibilities in the future of the types of clinical trials that JOHD subjects may be included in [7].

The entire HD community—patients, family members at-risk for HD, caregivers, health-care professionals and scientists—has a keen interest in focusing attention on JOHD. There is a calling to better understand, and help, the plight of those that seem to have been “left behind” in the flurry of research studies on AOHD [8]. The study of patients who are afflicted early in life with HD has become an urgent need with this Special Issue representing just the beginning of the required effort. 
